# Effects of Relative Molecular Weight Distribution and Isoelectric Point on the Swelling Behavior of Gelatin Films

**DOI:** 10.3389/fchem.2022.857976

**Published:** 2022-05-26

**Authors:** Fangqi Ji, Wei Zhou, Ze Zhang, Bing Zhang

**Affiliations:** ^1^ School of Chemistry and Materials Engineering, Beijing Technology and Business University, Beijing, China; ^2^ Technical Institute of Physics and Chemistry, Chinese Academy of Science, Beijing, China

**Keywords:** Gelatin, film, molecular weight, swelling ratio, isoelectric point

## Abstract

The swelling behavior of gelatin films with different extraction processes are investigated. The results showed that the swelling ratio of the gelatin film extracted by alkaline hydrolysis of collagen (type-B) in a range of pH environments was higher than the one extracted by enzymatic hydrolysis collagen (type-E). In the drug releasing simulation, type-B gelatin capsules also showed a faster collapse rate than type-E gelatin capsules. Based on analyzing relative molecular weight distribution of type-B and type-E gelatins, the more widely distributed relative molecular weight is the key attribution for enabling easier diffusion of water molecules inside the porous channels of peptide chains. Furthermore, with the pH of solution environment far from the isoelectric point (pI) of gelatin films, the swelling ratios were found to increase remarkably, which is due to electrostatic repulsion expanding the pore size of peptide chains. Finally, the addition of SO_4_^2−^ in gelatin film was performed to confirm the dominant effect of component compared to pI on swelling behavior of gelatin films.

## 1 Introduction

Gelatin is a kind of natural macromolecular compound, which belongs to the hydrocolloid family and can be extracted from skin, bone and connective tissues of animals through partial hydrolysis of collagen (Jridi et al., 2013; Dara et al., 2020). Different preparation techniques can lead to the difference in relative molecular weight distribution of gelatin (Sittiruk and Siriporn 2006; Ahmad et al., 2018). The relative molecular weight distribution of gelatin is mainly concentrated in about 100, 200 and 300 kg/mol, corresponding to *α*, *β*, and *γ* peptide chains, respectively (Tu et al., 2015). In addition, there are other types of gelatin with either lower relative molecular weight or higher relative molecular weight. Based on different extraction processes, gelatin can be mainly grouped into type-B and type-E. The type-B gelatin is extracted by alkaline hydrolysis of collagen, with higher rigidity and wider relative molecular weight distribution. The type-E gelatin is extracted by enzymatic hydrolysis collagen, with higher elasticity and relatively narrow relative molecular weight distribution (Ahmad et al., 2017). Corresponding to the hydrolysis methods, the isoelectric points of type-B and type-E gelatin are 4.6–5.2 and 7.0–9.0, respectively (Gómez-Guillén et al., 2011).

Functional properties of gelatin, such as freezing strength, viscosity and gel strength, are highly associated with its relative molecular weight distribution (Elharfaoui et al., 2007). The bloom number of gelatin corresponds to the proportion of *α* chain, and the greater the proportion of *α* chain, the greater the gel strength of gelatin (Netter et al., 2020). The viscosity of gelatin is related to its relative molecular weight, and the higher high relative molecular weight components, the higher the viscosity (Enrione et al., 2020). The gel strength of gelatin mainly depends on its molecular properties, especially relative molecular weight distribution and amino acid composition (Gómez-Guillén et al., 2002). The greater content of high relative molecular weight components corresponds to the higher gelation properties of gelatin. Therefore, different extraction processes can lead to different relative molecular weights of gelatin, as well as different physical and structural properties of gelatin (Muyonga et al., 2004; Carvalho et al., 2008).

Gelatin has been widely used in biomedicine due to its high biocompatibility and low cost (Rubini et al., 2020). With no toxicity and enhanced absorption of medicine by human body, gelatin is a favorable raw material used in medical packaging films (Madkhali et al., 2019). Drugs can be loaded in gelatin films through physical adsorption or embedding, and drug-loaded gelatin films can be swollen and broken at different pHs to release drugs (Marín et al., 2018). As one of the important characteristics of gelatin film, its swelling capacity is sensitive to temperature and pH in various environments (Peter et al., 2010; Pinto Ramos et al., 2019). Recently, many studies have focused on swelling properties of gelatin film (Zhang et al., 2020). For example, quercetin is loaded on gelatin films to modulate their mechanical and swelling properties (Rubini et al., 2020), the addition of chitosan can increase the thickness of gelatin films but decrease the swelling ratio (Mano et al., 2017), and the addition of cross-linking agents can decrease the swelling ratio of gelatin-based composite films (Kunal et al., 2010; Alina et al., 2020). Bukhari examined the effect of poly molecular weight and concentration in a gelatin-PEO semi-interpenetrating polymer network on the swelling behavior and enzyme-induced degradation in simulated gastric fluid and simulated intestinal fluid at 37°C (Bukhari et al., 2015). Current research on swelling properties has focused on the addition of exogenous additives to modify swelling properties and investigate the associated changes in properties, for example, the addition of quercetin, chitosan, cross-linking agents, etc. The research on the relative molecular weight of gelatin on swelling properties is missing.

The aim of this work was to investigate effects of relative molecular weight distribution and isoelectric point on the swelling properties of gelatin films. Firstly, we selected several typical enzymatic gelatins and alkaline gelatins for the study. The solvent-casting method was used to prepared gelatin films with different types of gelatin. Physicochemical properties of films were determined by different experimental techniques such as fourier transform infrared, thermogravimetric analysis and differential scanning calorimetry. Analyzed the responsiveness of gelatin films swelling to the relative molecular weight composition of gelatin raw materials. Furthermore, we also studied the swelling properties of gelatin films by changing the pHs of the solution and adding SO_4_^2−^.

## 2 Materials and Methods

### 2.1 Materials

Type-B gelatin was purchased from Dongbao Bio-tech Co., Ltd. (Baotou, China), and type-E gelatin was purchased from Ningxia Xinhaoyuan Biotechnology Co., Ltd. (Ningxia, China). Other chemicals, including hydrochloric acid (HCl), sodium sulfate (Na_2_SO_4_), sodium hydroxide (NaOH), sodium dihydrogen phosphate (NaH_2_PO_4_), disodium hydrogen phosphate (Na_2_HPO_4_), sodium chloride (NaCl), polyethylene glycol (PEG), Rhodamine B and talcum powder, were purchased from Sinopharm Chemical Reagents Co., Ltd. (Shanghai, China). All chemicals were of analytical grade and used without further purification.

### 2.2 Methods

#### 2.2.1 Preparation of Gelatin Film

Gelatin films were prepared using the solvent-casting method (Leite et al., 2021). Briefly, 2.5 g gelatin was soaked in 50 ml deionized (DI) water at room temperature for 20 min, and then heated at 40°C with stirring until gelatin was completely dissolved. A certain volume of gelatin solution (15 ml) was cast on an acrylic sheet (10 × 10 cm^2^), dried at 35°C for 24 h and peeled off the sheet. Similarly, five types of gelatin films were prepared by B1, B2, B3, E1, and E2 gelatins as shown in Table 1, respectively. In addition, Na_2_SO_4_ (0.25 g) was added into the gelatin solution (15 ml) to prepare an inorganic ion composite film (B2S film).

**TABLE 1:** Physicochemical properties of different types of gelatin used in this study. Film thickness and PD were taken as average values.

Product number	Sample	Extraction method	Film thickness (μm)	PD	pI
B0025	B1	Alkaline	5.75 ± 0.21	2.772 ± 0.0005	5.0
B0209	B2	Alkaline	5.85 ± 0.31	2.758 ± 0.0003	4.5
B20171028	B3	Alkaline	5.77 ± 0.41	2.698 ± 0.0021	4.8
Y170117	E1	Enzymatic	5.80 ± 0.39	2.360 ± 0.0032	7.0
S180205	E2	Enzymatic	5.85 ± 0.38	2.455 ± 0.0010	7.5

#### 2.2.2 Preparation of Gelatin Capsules

Gelatin capsules are typically prepared with the dipping glue method (Fauzi et al., 2021). Gelatin (7.0 kg) was added to deionized water (19 kg, 75°C) and stirred at 55°C until gelatin was completely dissolved. The solution was defoamed in a vacuum environment, where the vacuum was −0.09 MPa. Then the gelatin solution was then kept for 2 h under normal pressure. The gelatin solution (24 MPa s) was poured into a container, and the viscosity of the gelatin solution was kept at 55°C. Capsules were prepared by dipping mould pins into the gelatin solution. The mould pins were rotated to be shaped at room temperature, dried in hot air of 30°C for 2.5 h. The dried capsules were removed from the molds and stored at room temperature for use.

### 2.3 Characterization

#### 2.3.1 Relative Molecular Weight Distribution of Gelatin

The relative molecular weight distribution of different types of gelatin was determined using size-exclusion chromatography coupled with miniDAWN multi-angle light scattering (SEC-MALS, Wyatt Technology Corp., Santa Barbara, CA, United States). The mobile phase was prepared by mixing 25 mM sodium dihydrogen phosphate, 25 mM disodium hydrogen phosphate and 50 mM sodium chloride. Here, Milli-Q ultrapure water was filtered through a 0.22-μm filter before use. The gelatin solution was prepared using the mobile phase at a concentration of 6 mg/ml, and kept at room temperature for 12 h prior to use. The columns and pipelines were washed with the mobile phase before measurements. When the laser signal fluctuation was less than 0.05%, the instrument was calibrated by injecting 2 mg/ml PEG (MW = 10000). The injection volume was 200 μL, and the flow rate was 0.5 ml/min. The column (Ohpak SB-806 HQ 300 × 8 mm) outlet was connected to a multi-angle laser light scattering photometer followed by a differential refractometer. The column was operated at 40°C, which was higher than the melting point of gelatin. The measurements were repeated at least three times.

The number-average molecular weight (Mn) and weight-average molecular weight (Mw) were calculated using ASTRA software (version 5.3.4.14, Wyatt, United States). The polydispersity (PD) of different types of gelatin was calculated using Eq. 1:

$$PD=\frac{Mw}{Mn}$$ (1)

#### 2.3.2 Determination of Functional Groups

Fourier Transform Infrared (FTIR) Spectroscopy of gelatin films were recorded on a Nicolet™ iS™10 FTIR spectrometer (Thermo Fisher Scientific, Massachusetts, United States). Briefly, 1 mg dry gelatin was ground into powder for infrared scanning, and the gelatin film (1 × 1 cm^2^) was directly scanned. Each spectrum was collected after 32 scans in the range of 4,000–450 cm^−1^ at a resolution of 4 cm^−1^.

#### 2.3.3 Thermal Stability of Films

The thermal stability of the sample was studied by the Simultaneous Thermal Analyzer (STA) (Netzsch, Germany). The Thermogravimetric Analysis (TGA) and Differential Scanning Calorimetry (DSC) analysis were performed from room temperature to 800°C at 10 K/min under a nitrogen atmosphere.

#### 2.3.4 Isoelectric Point of Gelatin Film

The isoelectric points (pI) of B1, B2, E1, E2, and B2S gelatin films were determined as reported previously (Wang et al., 2016; Ji et al., 2020). For example, 0.5 g of gelatin film was dissolved in a 10 ml aqueous solution with a certain pH, and then was heated at 40°C for 15 min. The gelatin solution was placed into a colorimetric tube and then cooled in ice-water bath until frozen. The above experiment was repeated for each kind of gelatin film with the only variable pH of aqueous solution from 4.0 to 9.0. Based on absorbance test results, the pH of the gelatin solution with minimum transparency was confirmed as the pI of the corresponding gelatin film.

#### 2.3.5 Swelling Ratio of Gelatin Film

The swelling ratio (SR) of type-B, type-E, and B2S gelatin films were measured by weighing method. At room temperature, a certain amount of dry gelatin film was placed in 20 ml of water for a certain period. Then took the film out and removed water with filter paper, the swollen gelatin films were weighed again. The swelling ratio of gelatin films was calculated using Eq. 2:

$$SR\left(\%\right)=\frac{W_{t}-W_{o}}{W_{o}}\times 100$$ (2)

where *W*_o_ is the weight of the dry gelatin film before swelling (g), and *W*_t_ is the total weight of swollen gelatin films (g). Similarly, the swelling ratios of different gelatin films (B1, B2, E1, E2, and B2S) at different pHs of 2, 5.6, and 8 were calculated. The pH of the solution was adjusted by adding HCl or NaOH, and the pH was measured by a pH meter [Mettler Toledo International Trading (Shanghai), Shanghai, China].

#### 2.3.6 Disintegration Test of Capsules

Talcum powder and rhodamine B were used to simulate the release of drugs from gelatin capsules. Briefly, 33 mg of rhodamine B and 18 g of talc powder were added in 10 ml of DI water, mixed and freeze-dried to obtain a mixture of talcum powder and rhodamine B. Six gelatin capsules were weighed and loaded with the mixture. After the residual mixture on the surface were wiped off, the capsules were weighed again. Drug-loaded gelatin capsules were added in ZB-3A fully automatic disintegration tester (TIANDDA TIANFA pharmaceutical testing instrument manufacturer, Tianjin, China) containing 770 ± 10 g of DI water at 37 ± 1°C. During disintegration, 5 ml of solution was sampled every 60 s, followed by the addition of another 5 ml of DI water in the tube. The absorbance of the sample was measured and the dissolution concentration of simulated drugs from gelatin capsules were calculated according to the standard curve of Rhodamine B absorbance, and then the dissolution rate was calculated.

## 3 Results and Discussion

### 3.1 Relative Molecular Weight Distribution Analysis

**FIGURE 1:**
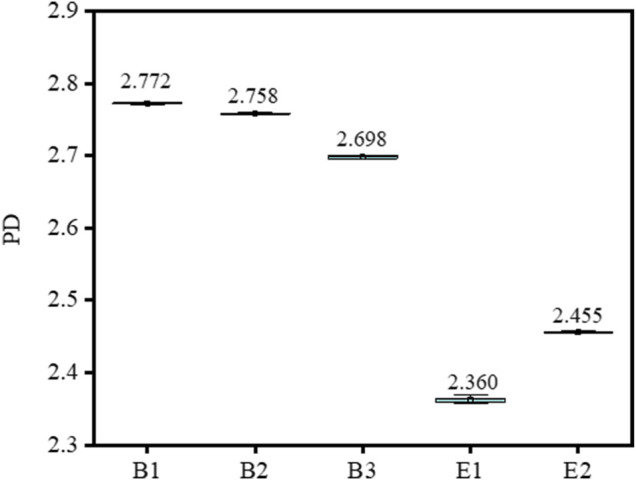
The polydispersity (PD) of different types of gelatin.

The PD of different types of gelatin are shown in Figure 1 and Table 1. The PD of gelatin was calculated as the ratio of M_w_/M_n_, and the specific values of M_w_ and M_n_ are shown in Supplementary Table S1. The larger the PD, the broader the relative molecular weight of gelatin. It can be seen that the PD of type-B gelatin was remarkably larger than those of type-E gelatin, and the relative molecular weight distribution of type-B gelatin could be confirmed to be broader than that of type-E gelatin in this research.

### 3.2 Determination of Functional Groups

**FIGURE 2:**
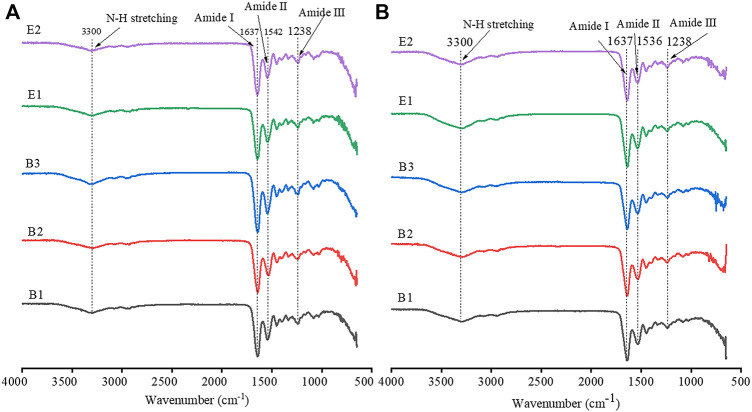
Fourier Transform Infrared (FTIR) spectra of gelatin **(A)** and gelatin film **(B)**.

FTIR spectra of different types of gelatin and gelatin films are shown in Figure 2. The FTIR spectra of both gelatin and gelatin film showed four characteristic peaks of collagen, including amide A, amide I, amide II, and amide III (Wang et al., 2001; Deng et al., 2020). The peaks of amide A were attributed to the stretching vibration of N-H groups, which generally occurs at 3300 cm^−1^ (Wang et al., 2001). The C=O stretching vibration of amide I occurred at 1637 cm^−1^, the C-N or N-H stretching vibration of amide II occurred at 1540 cm^−1^, and the characteristic absorption peak of amide III occurred at 1238 cm^−1^. Type-B gelatin and type-E gelatin showed similar FTIR spectra, and FTIR spectra of gelatin films were consistent with those of gelatin. The results suggested that the functional groups composition of type-B and type-E gelatin and gelatin films were basically the same. In addition, the preparation of films had no impact on the chemical bond of gelatin.

### 3.3 Thermal Stability of Films

**FIGURE 3:**
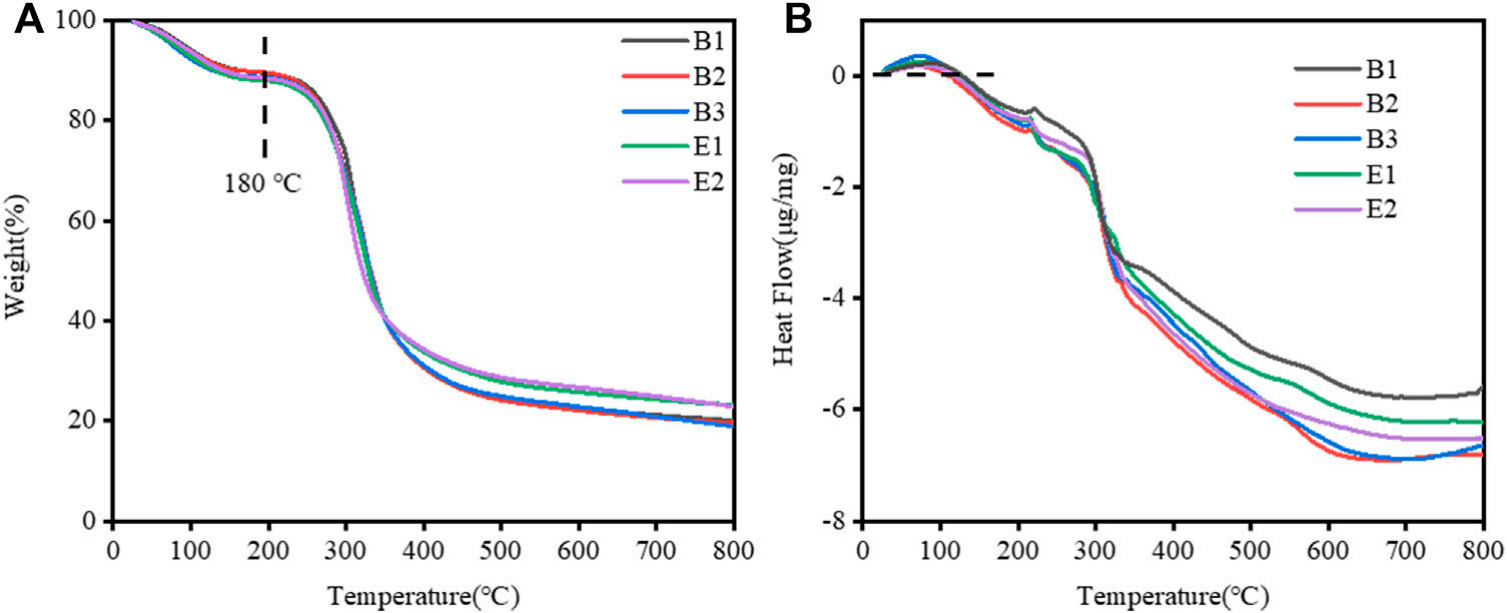
**(A)** Thermal gravimetric analysis (TGA) and **(B)** Differential Scanning Calorimetry (DSC) curves of different types of gelatin films.

The thermal decomposition of gelatin films was evaluated, as shown in Figure 3A which showed two thermal processes. First, an endothermic process occurred between the room temperature to 180°C with an initial weight loss of about 9.00% which is attributed to the evaporation of water. The second process was an endothermic process observed between 180 and 800°C, corresponding to a mass loss attributed to the thermal decomposition of gelatin. The weight loss data for the two processes are shown in Table 2. The close weight loss of all gelatin films in the first phase indicates that their water content is close.

**TABLE 2:** Data of Thermal gravimetric analysis (TGA)/Differential Scanning Calorimetry (DSC).

Sample	Weight (%) (−180°C)	Weight (%) (180–800°C)	T_max_ (°C) (Melting transition temperature)
B1	8.92	71.04	87.46
B2	8.98	71.32	82.49
B3	8.80	72.34	84.02
E1	9.11	68.77	86.08
E2	8.96	69.26	84.39

DSC thermograms of all of the films studied are detailed in Figure 3B. The gelatin films exhibited a T_max_ (melting transition temperature, endothermic peak) in Table 2. The T_max_ of different gelatin films are similar.

Therefore, we found that the water content, T_max_, and thickness (in Table 1) of different types of gelatin films are similar. They have little effect on the differences in the swelling properties of different types of gelatin films.

### 3.4 Swelling Ratio

#### 3.4.1 Effect of Relative Molecular Weight Distribution

Since the swelling properties of each type of gelatin are similar, we took pictures of the swelling behavior of one of each type. The swelling behavior of gelatin films in pH 5.6 are shown in Supplementary Figure S1, in which the boundaries of films were outlined with dashed lines. With similar initial area, the swelling area of type-B gelatin film was significantly higher than that of type-E gelatin film at specific time points. When the swelling time increased to 240 min, the area of B2 film is almost 1.5 times of E2 film. For a more visual representation, we added the corresponding area of swollen gelatin film. The area of swollen gelatin film (type B and type E) in water at pH 2 for 45 min are shown in Supplementary Table S2. The area of swollen gelatin film (type B and type E) in water at pH 5.6 for 240 min in Supplementary Table S3. The area of swollen gelatin film (type B and type E) in water at its corresponding pI for 240 min in Supplementary Table S3. Each gelatin film has the smallest change in swelling area at its isoelectric point, and the smaller PD of each gelatin film, the smaller the change in swelling area.

**FIGURE 4:**
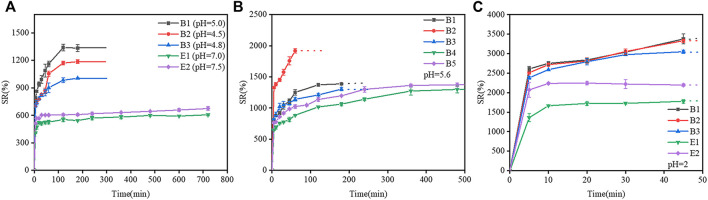
Swelling curve of different gelatin films. **(A)** At the pH of the gelatin films isoelectric point, **(B)** At pH 5.6, **(C)** At pH 2 (the dashed lines indicate the samples dissolved in the water and cannot be observed).

The swelling curves of gelatin films at different pH are shown in Figure 4. In Figures 4A,B,C, the swelling ratios of type-B gelatin films were remarkably higher than those of type-E gelatin films. The swelling ratios of gelatin (B1, B2, B3, E1, and E2) films increased rapidly over time within 100 min, then gelatin films were gradually increased until the swelling equilibrium dissolved and disappeared in Figure 4A. In Figure 4B, The swelling ratios of B1 film increased rapidly over time within 60 min, the swelling ratios of B2, B3, E1, and E2 films increased rapidly over time within 200 min, then gelatin films were gradually increased until the swelling equilibrium dissolved and disappeared In Figure 4C, B1, B2, B3, E1, and E2 films swell rapidly within 45 min and start to dissolve at 45 min. In conclusion, the trend of all swelling ratios is basically consistent with the change of swelling area.

The extraction process of type-B gelatin could destroy the helical structure of collagen and cleave the cross-links between molecules, leading to a broad relative molecular weight distribution of type-B gelatin. However, the relative molecular weight distribution of type E gelatin was narrow, and the integrity of the helical structure of collagen was high. Therefore, water molecules can easily penetrate and diffuse into type-B gelatin, leading to the rapid swelling of gelatin. And at the same time, the intermolecular force in gelatin films decreased gradually over time, leading to the relaxation of polypeptide chains and the dissolution of gelatin. In contrast, water molecules can only permeate and diffuse slowly into type-E gelatin, leading to the relatively slow swelling rate of gelatin. And the swelling of type-E gelatin film was slower than that of type-B gelatin film in a certain period of time, during which type-E gelatin film was not dissolved in water. Therefore, the relative molecular weight distribution could affect the swelling of gelatin film.

**FIGURE 5:**
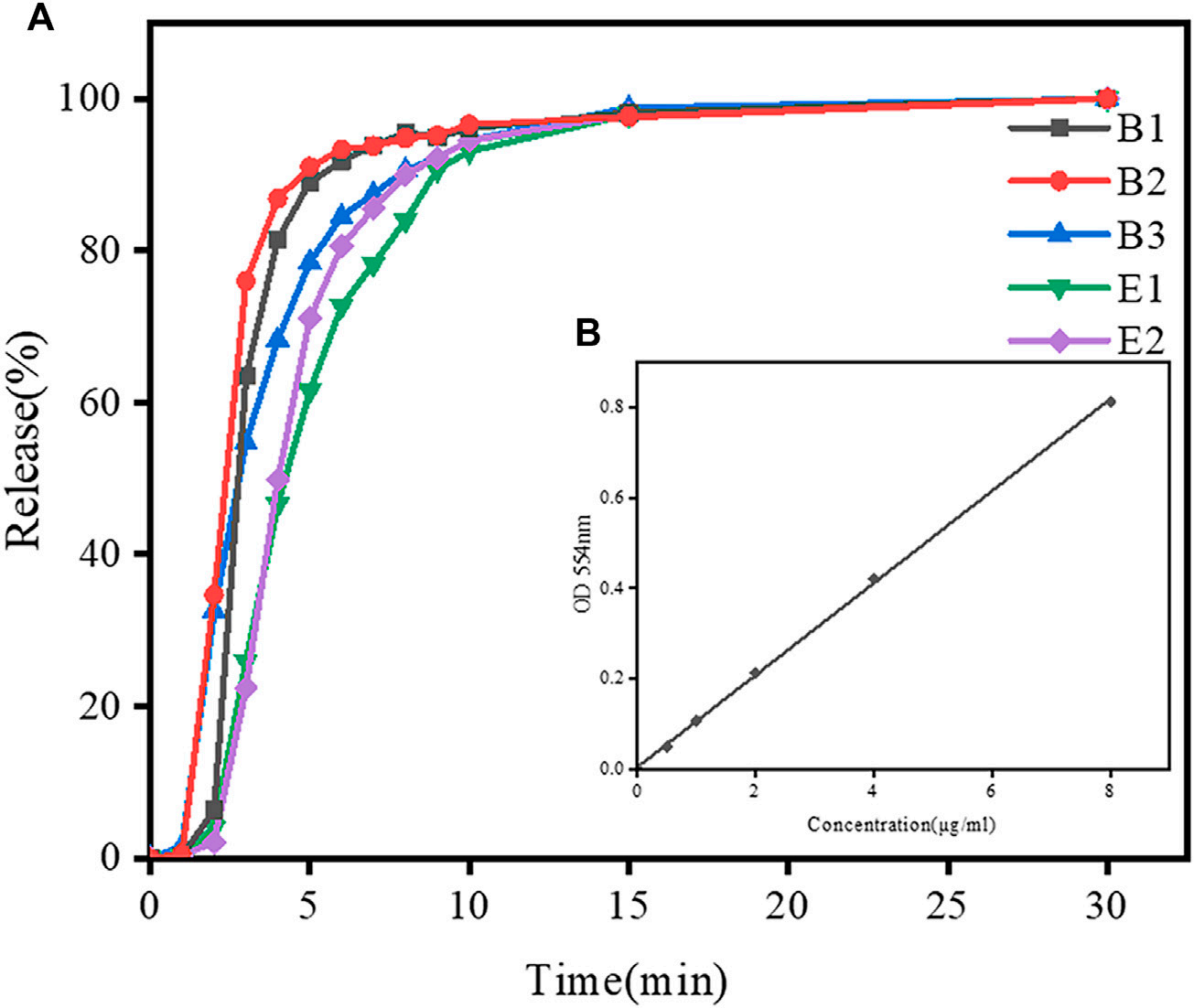
Dissolution profiles of simulated drugs from different gelatin capsules within 30 min in disintegration test in DI water. **(A)** Drug release curves of B2 and E2 capsules in disintegration test. **(B)**The standard curve of Rhodamine B absorbance.

The morphology of gelatin capsules (type B and E) with and without loading of Rhodamine B mixture is shown in Supplementary Figure S2. Two gelatin capsules have similar morphology. In order to better compare the swelling behavior of type-B and type-E gelatin, disintegration test of gelatin capsules at pH 5.6 was conducted. The standard curve of Rhodamine B absorbance is shown in Figure 5B. During the disintegration test, the absorbance of the sample was measured and the dissolution concentration of simulated drugs from gelatin capsules were calculated according to the standard curve. The release curve of a simulated drug from gelatin capsules in disintegration test is shown in Figure 5A. In the initial 10 min, the release rate of drug from type-B capsules was higher than that from type-E capsules. After 10 min, most of loaded drugs were released from both types of capsules. The result suggested that the transition of type-B gelatin film from swelling to dissolution was faster than that of type-E gelatin film. It is consistent with the change of swelling ratios and all capsules meet the pharmacopoeia regulations.

#### 3.4.2 Effect of Isoelectric Points

pI is the pH at which there is no charge on the surface of a molecule (Lautenbach et al., 2021). Due to the presence of different amino acid residues, proteins have different isoelectric points (Hitchcock 1929). At isoelectric points, gelatin chains are in the most coiled conformation, and gelatin can be temporarily flocculated, appearing milky white under the light. The isoelectric point could affect the swelling and dissolution of gelatin films, thereby affecting the controlled release of drugs from gelatin capsules and the degradation of gelatin films used in food packaging (Congde et al., 2012; Kavoosi et al., 2013; Pour et al., 2019).

**FIGURE 6:**
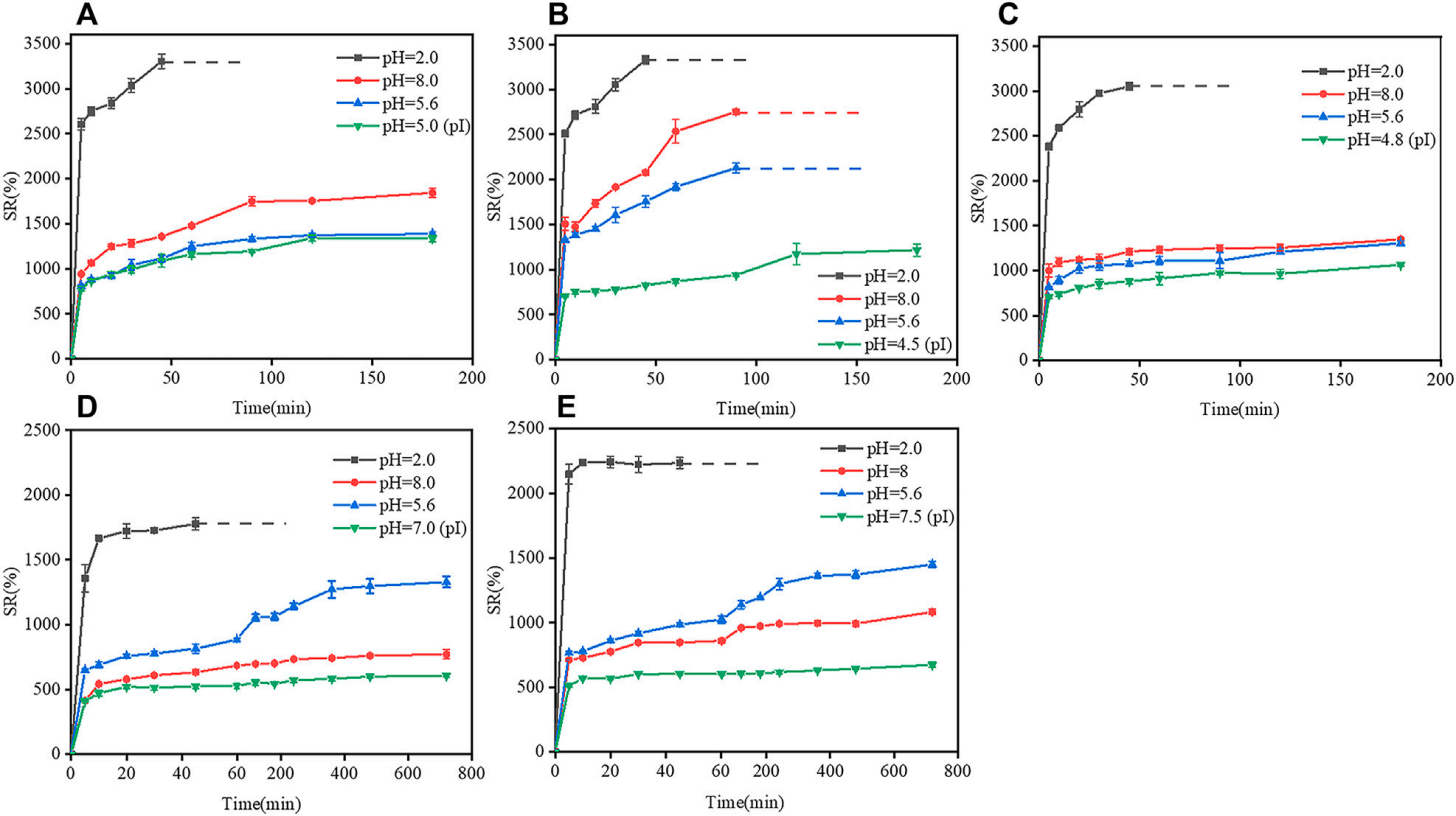
Effects of isoelectric points on the swelling ratio of gelatin films at different pHs. **(A)** B1 film, **(B)** B2 film, **(C)** B3 film, **(D)** E1 film, and **(E)** E2 film. Dotted lines indicate that gelatin films were completely dissolved in water.

The swelling response of two types of gelatin (B1, B2, B3, E1, and E2) with different pIs (Table 1) to different pHs (2, 5.6, 8, and pI of the corresponding film) are shown in Figure 6. At pH 2, B1 film reached a maximum swelling ratio of 3400% after 45 min of immersion in water, and was then completely dissolved in water. At pHs of 5.6, 5.0 (pI) and 8, the swelling ratio of B1 film increased with time in 0–120 min; a swelling equilibrium was reached in 120–180 min; and B1 film was dissolved at 180 min. The result indicated that the swelling ratio of B1 film was low when the pH was close to the isoelectric point, and at the isoelectric point, the dissolution rate is minimal, but its swelling ratio was high when the pH was far from the isoelectric point. The changes in the swelling rates of B2, B3, E1, and E2 films were similar to that of B1 film.

The phenomenon could be explained as follows. At pHs close to the pI, the polypeptide chains could be collapsed due to the electrostatic attraction between the opposite charges, which caused the contraction of gelatin chains, the decrease in the surface porosity and swelling ratio of gelatin films; however, at pHs far from the pI, gelatin molecules were protonated and charged, and electrostatic repulsion could extend polypeptide chains and increase the swelling ratios of gelatin films (Hegab et al., 2020; Ozcan and Cagil 2020). In either type-B gelatin or type-E gelatin, the changes in the pH of the solution could cause the protonation/deprotonation of polypeptides molecules, leading to charge repulsion and conformational change of gelatin chains, which further affected the swelling properties of gelatin films (Chen et al., 2019).

#### 3.4.3 Effects of Additional Ionic Group

**FIGURE 7:**
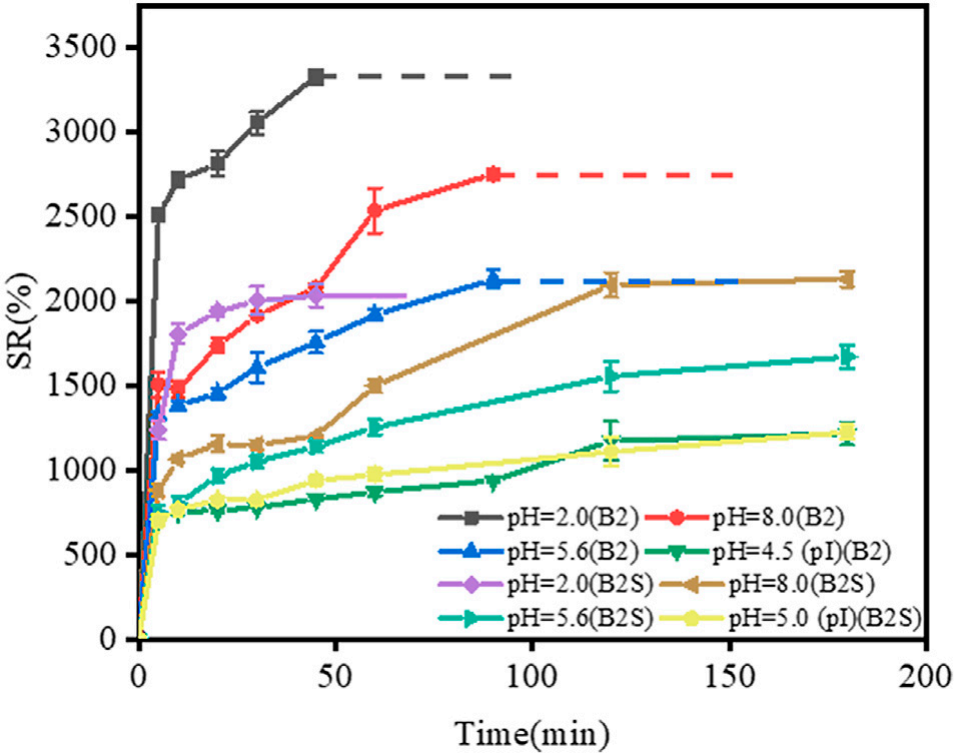
Swelling curve of gelatin films (B2 and B2S) at pHs of 2, 5.6, 8, and the pH of the gelatin films pI. Dotted lines represent the dissolution of gelatin films in water. In B2S film, gelatin: SO_4_^2−^ = 10:1 (g/g).

As shown in Figure 7, the swelling ratio of B2 film was higher than that of B2S film at pHs of 2, 5.6, and 8, B2 films were dissolved earlier than B2S films. In addition, the pI of B2S film was 5, it is expected that its swelling ratio was much lower at the pI than at pH 2.0, pH 5.6 and pH 8, however, the difference of swelling ratio was significantly smaller than that of B2 films. The result show that the effect of SO_4_^2−^ on swelling behavior of composite films is greater than that of deviating from pI. The introduction of SO_4_^2−^ might change the pI of B2 film, and the shielding effect of SO_4_^2−^ could decrease the swelling ratio of gelatin by forming a more compact structure of gelatin film. Furthermore, the result suggested that the addition of SO_4_^2−^ could regulate the swelling ability of gelatin films at different pHs, which can be better utilized in drug release and targeted therapy (Frutos et al., 2010).

## 4 Conclusion

In this paper, the effect of the difference of relative molecular weight distribution between type-B gelatin and type-E gelatin on the related swelling properties was studied. The results showed that at pH = 2, pH = 5.6 and pH = pI, the swelling ratio of B type gelatin film was higher than E type gelatin film, which was attributed to higher polydispersity coefficient and wider relative molecular weight distribution of B type gelatin film than E type gelatin film. The water molecules are more easily diffused into the gelatin film, and the swelling is more remarkable. It was found that the dissolution rate of type-B gelatin capsule was greater than that of E type gelatin capsule in disintegration test. In order to study the effect of isoelectric point on the swelling properties of gelatin film by setting the pH value of external environment, we found that the swelling ratio of gelatin film decreases when the pH value of environment is close to the isoelectric point. When it is far from the isoelectric point, the expansion of the peptide chain can attract more water molecules, and the swelling ratio of the membrane increases. The swelling ratio of gelatin film was changed by adding SO_4_^2−^, we performed to confirm the dominant effect of component compared to pI on swelling behavior of gelatin films. By regulating the swelling properties of gelatin film, gelatin film had better ability to adapt to acid-base environment, which provided experimental basis for improving the selection of biomedicine gelatin-based materials. The results offered constructive information based on intrinsic factors for the choice of gelatin raw materials in different application fields. On the basis we can further optimise performance by choosing the best combination of raw materials, such as the swelling properties of gelatin hard capsules, the water solubility of packaging materials.

## Data Availability

The original contributions presented in the study are included in the article/Supplementary Material, further inquiries can be directed to the corresponding authors.
