# Supplementary material for: Effects of Relative Molecular Weight Distribution and Isoelectric Point on the Swelling Behavior of Gelatin Films
Source: Front Chem. 2022 May 26;10:857976. doi: 10.3389/fchem.2022.857976 (PMC9178206; doi:10.3389/fchem.2022.857976)
Supplement: Supplementary file 1 [file DataSheet1.docx]

Supplementary Material for Effects of molecular weight distribution and isoelectric point on the swelling behavior of gelatin films


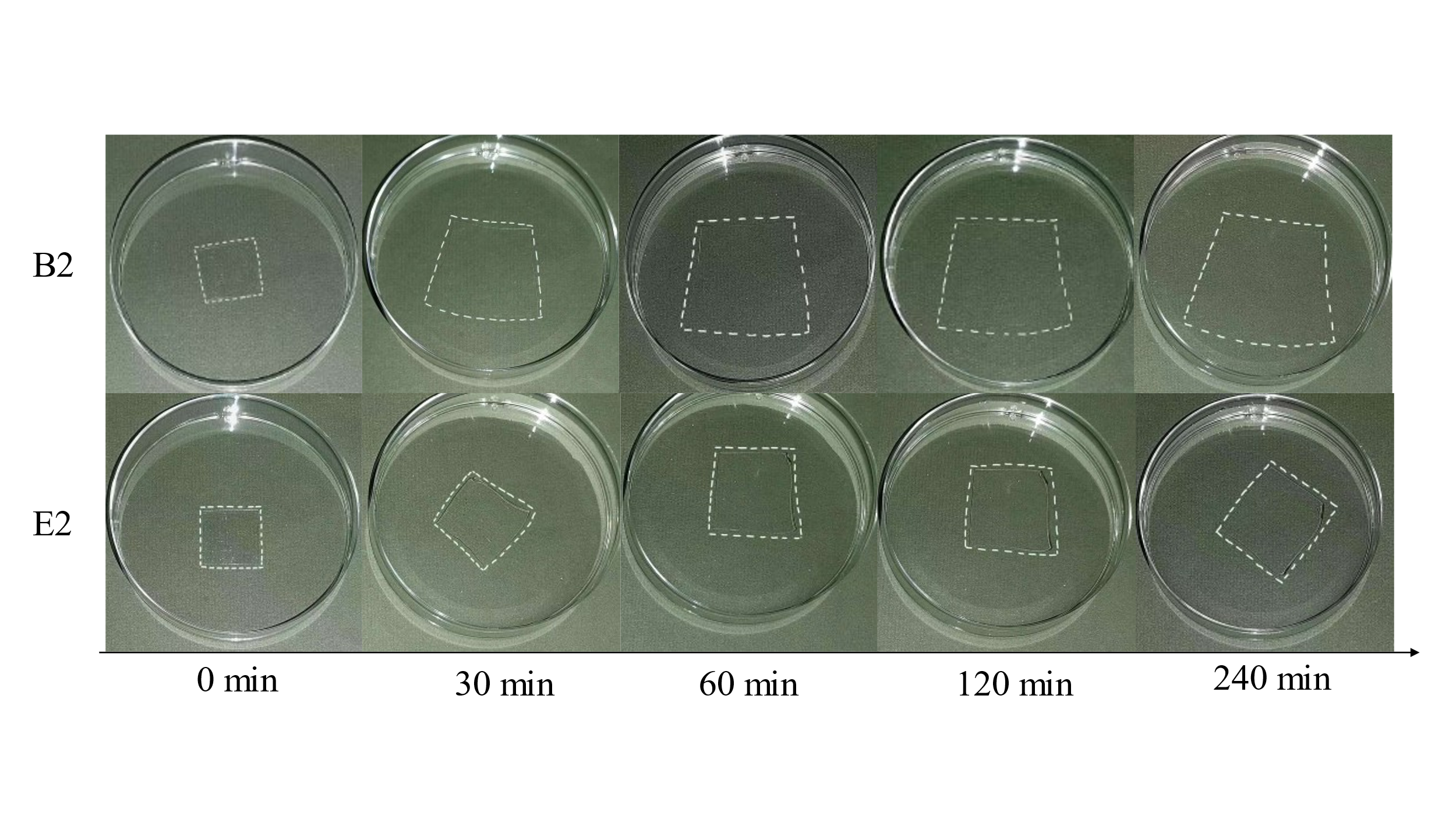


**Figure S1** Photographs of swollen gelatin films (type B and type E) in DI water at pH 5.6 within 240 minutes


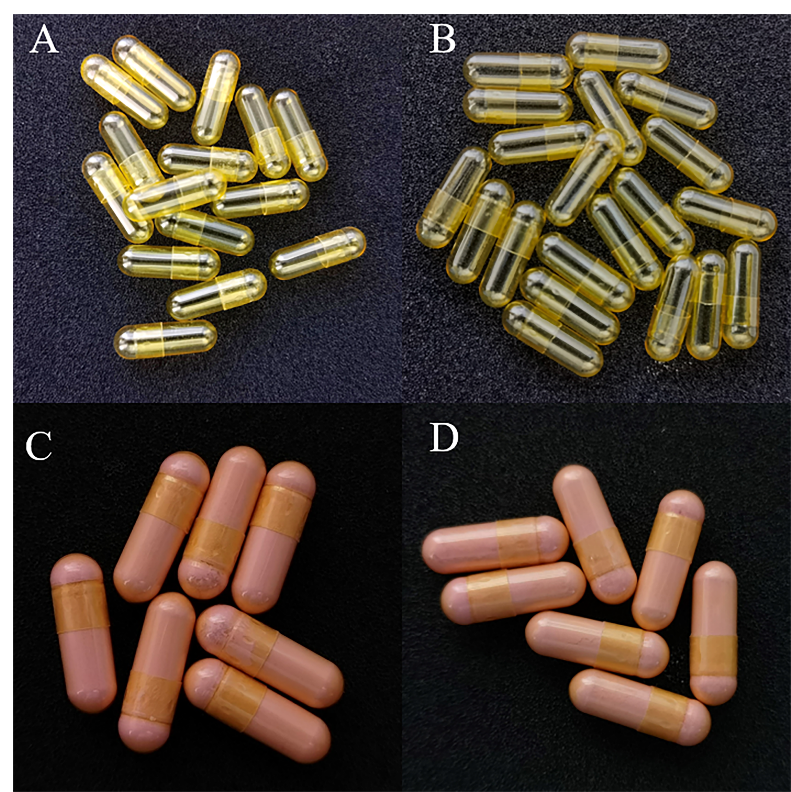


**Figure S2** Photographs of gelatin capsules. (A) Type B without mixture, (B) Type E without mixture, (C) Type B loading mixture, and (D) Type E loading mixture

Table S1 Molar mass moments of different types of gelatin determined by SEC-MALLS

| **Sample** | **M_w_ (kg/mol)** | **M_n_ (kg/mol)** |
| --- | --- | --- |
| B1 | 179.80±0.0058 | 64.88±0.0290 |
| B2 | 191.10±0.0001 | 69.30±0.02512 |
| B3 | 187.40±0.0057 | 69.47±0.0521 |
| E1 | 171.90±0.0755 | 72.84±0.0671 |
| E2 | 145.60±0.0404 | 59.30±0.0129 |

Table S2 Area of swollen gelatin film (type B and type E) in water at pH 2 for 45 minutes

| **Time (min)** | **B1 (cm^-2^)** | **B2 (cm^-2^)** | **B3 (cm^-2^)** | **E1 (cm^-2^)** | **E2 (cm^-2^)** |
| --- | --- | --- | --- | --- | --- |
| **0** | 4 | 4 | 4 | 4 | 4 |
| **5** | 30.25 | 28.09 | 25 | 7.84 | 18.06 |
| **10** | 34.81 | 33.64 | 32.49 | 14.06 | 26.01 |
| **20** | 36 | 36 | 34.81 | 14.44 | 27.56 |
| **30** | 39.69 | 40.32 | 37.21 | 14.82 | 28.09 |
| **45** | 42.25 | 42.3801 | 39.68 | 15.6 | 28.196 |

**Table S3.** Area of swollen gelatin film (type B and type E) in water at pH 5.6 for 240 minutes

| **Time (min)** | **B1 (cm^-2^)** | **B2 (cm^-2^)** | **B3 (cm^-2^)** | **E1 (cm^-2^)** | **E2 (cm^-2^)** |
| --- | --- | --- | --- | --- | --- |
| **0** | 4.00 | 4.00 | 4.00 | 4.00 | 4.00 |
| **5** | 11.40 | 11.56 | 10.89 | 5.29 | 10.89 |
| **10** | 13.65 | 12.96 | 12.25 | 5.75 | 11.56 |
| **20** | 16.00 | 16.00 | 12.60 | 6.16 | 11.83 |
| **30** | 16.40 | 16.81 | 13.69 | 6.48 | 12.04 |
| **45** | 17.02 | 17.01 | 14.06 | 6.88 | 12.25 |
| **60** | 17.22 | 17.30 | 14.44 | 7.36 | 12.67 |
| **90** | 17.64 | 17.43 | 14.82 | 7.54 | 12.78 |
| **120** | 17.89 | 17.56 | 15.60 | 7.911 | 12.85 |
| **180** | 18.49 | 17.64 | 16.00 | 7.96 | 12.96 |
| **240** | 18.75 | 17.72 | 16.81 | 8.12 | 13.18 |

**Table S4.** Area of swollen gelatin film (type B and type E) in water at its corresponding pI for 240 minutes

| **Time (min)** | **B1 (cm^-2^)** | **B2 (cm^-2^)** | **B3 (cm^-2^)** | **E1 (cm^-2^)** | **E2 (cm^-2^)** |
| --- | --- | --- | --- | --- | --- |
| **0** | 4.00 | 4.00 | 4.00 | 4.00 | 4.00 |
| **5** | 10.89 | 9.61 | 9.00 | 5.06 | 8.70 |
| **10** | 12.96 | 11.29 | 10.24 | 5.52 | 9.00 |
| **20** | 13.32 | 11.59 | 10.59 | 6.13 | 9.61 |
| **30** | 13.84 | 12.25 | 11.22 | 6.25 | 9.92 |
| **45** | 15.21 | 12.96 | 11.55 | 6.50 | 10.05 |
| **60** | 16.81 | 13.14 | 11.90 | 7.02 | 10.14 |
| **90** | 17.14 | 13.32 | 12.11 | 7.15 | 10.21 |
| **120** | 17.39 | 14.06 | 12.25 | 7.40 | 10.30 |
| **180** | 17.56 | 14.74 | 12.43 | 7.48 | 10.56 |
| **240** | 17.64 | 15.21 | 12.50 | 7.78 | 10.63 |
